# Improving Incoming Resident (Foundation Year 1 and Foundation Year 2) Doctor Confidence in Starting a Haematology Placement Through a Structured Handbook

**DOI:** 10.7759/cureus.96648

**Published:** 2025-11-12

**Authors:** Syeda Liaba Hassan

**Affiliations:** 1 Haematology, The Royal Wolverhampton NHS Trust, Wolverhampton, GBR

**Keywords:** foundation year doctor, haematology, induction handbook, medical resident education, pdsa cycle, plan-do-study-act (pdsa), quality improvement research, resident doctor

## Abstract

Background: Junior doctors often report low confidence when starting haematology placements due to limited prior exposure and the complexity of the specialty.

Objective: The objective of this study was to improve preparedness and confidence among Foundation Year 1 (FY1) and Foundation Year 2 (FY2) doctors by introducing a structured haematology placement handbook.

Methods: A digital handbook summarizing key ward routines, documentation standards, transfusion procedures, and discharge pathways was created and distributed to incoming residents. Two Plan-Do-Study-Act (PDSA) cycles were conducted between December 2024 and July 2025, utilising pre-and post-intervention surveys using a five-point Likert scale to measure self-reported confidence in core domains. This was conducted in accordance with the SQUIRE 2.0 (Standards for Quality Improvement Reporting Excellence 2.0) guidelines.

Results: In Cycle 1, there were six participants with 50% pre-survey and 33% post-survey response rates. Mean confidence increased by +1.6 to +2.4 points across all domains. In Cycle 2, there were three participants with 100% pre-survey and 66% post-survey response rates. Mean confidence increase was +1.8 to +2.3 points across all domains. Qualitative feedback demonstrated improved preparedness, documentation, and understanding of ward tasks. All respondents recommended the handbook for future cohorts.

Conclusion: A structured haematology placement handbook significantly improved junior doctor confidence and preparedness. Integrating the handbook into departmental induction will help to enhance orientation and patient-care efficiency.

## Introduction

Transitioning into a new clinical speciality can be daunting for resident doctors, particularly in complex specialities such as haematology. Stress during this transition stems from managing responsibility, new clinical environments, and unfamiliar protocols [1]. Haematology is particularly challenging as it involves complex patient groups that can deteriorate quickly, with difficult-to-interpret laboratory results and complex coordinated care within the multidisciplinary team [2]. In the United Kingdom, medical students often have limited exposure to haematology as a speciality, resulting in varying levels of preparedness and confidence during placement induction.

Resident doctor confidence and preparedness are essential components of patient safety and clinical efficiency [3]. Low confidence in specialty-specific tasks may lead to delays in decision-making, increased dependence on senior clinicians, and reduced job satisfaction. As highlighted by NHS Education and Improvement Wales, an effective induction should aim to incorporate information essential for patient safety and should include practical information about the roles of the job [4]. This has been linked to improved junior doctor confidence, competence, and transition between placements [5].

Prior to the initiation of this quality improvement (QI) project, informal feedback was collected from resident doctors currently working in the department. Common challenges included ward task uncertainty, blood result interpretation, and management of deteriorating patients. This feedback highlighted the importance of a structured and supportive induction process. As a result, a digital induction handbook was conceptualised and developed with the aim of improving the quality, clarity, and consistency of departmental induction within the district general hospital. This report was developed with the SQUIRE 2.0 (Standards for Quality Improvement Reporting Excellence 2.0) framework in mind, ensuring a structured approach to all aspects of the QI cycle [6].

## Materials and methods

Study design

This was a QI project conducted from December 2024 to July 2025 in Russells Hall Hospital, Dudley, West Midlands, United Kingdom. The project aimed to improve resident doctors' preparedness and confidence while rotating into the haematology specialty. Each cycle of the QI project spanned a four-month period and coincided with the departmental rotation schedule. Incoming Foundation Year one (FY1) and Foundation Year two (FY2) doctors with no previous haematology experience were identified as key stakeholders for this project. 

The project's aim was defined and focused on project feasibility within the given timeframe. The ‘SMART’ (Specific, Measurable, Assignable, Realistic, Time-related) objectives were utilised to guide the development of suitable objectives [7]. The primary objective was to improve junior doctor confidence and reduce orientation-related confusion by creating and implementing a structured haematology placement handbook, with ≥80% of doctors reporting usefulness after use.

Intervention

A digital ‘Haematology Placement Handbook’ was developed to support resident doctors with induction to the haematology department. This handbook was distinct from a pre-existing departmental handbook that focused on haematology concepts and clinical knowledge. The new handbook concentrated on practical orientation and included detailed information about a typical ward day, approaches to prepping patient notes, and guidance on completing common ward jobs. The handbook also covered best practices for ward round documentation and offered advice on medication review and initiation relevant to resident doctors. Further sections focused on reviewing blood results, including full blood counts and tumour lysis syndrome panels, and outlined the procedures for requesting blood and platelet transfusions. Clear guidance was provided on admission documentation, clerking processes, discharge pathways, and writing discharge letters. The handbook also detailed how to initiate patient follow-up following discharge, listed useful departmental contacts, and provided an overview of weekly departmental activities.

In terms of handbook distribution, a digital PDF format was utilised for easy distribution to the incoming resident doctors. The handbook had embedded quick-response (QR) code links for pre-handbook and post-session surveys for evaluation of engagement and usability.

Plan-Do-Study-Act (PDSA) methodology

A PDSA methodology was developed to guide iterative testing of the project [8]. The framework was integrated throughout the project to ensure systematic refinement of the intervention based on ongoing feedback. In the ‘Plan’ phase, the intervention involved the development of a structured handbook for new incoming resident doctors. Pre-and post-intervention surveys were developed to assess self-reported confidence across eight key domains. Qualitative feedback on the handbook’s clarity and usefulness was also collected. In the ‘Do’ phase, the handbook was emailed to resident doctors two weeks into their rotation. This gave residents enough time to familiarize themselves with departmental workflows. The ‘Study’ phase involved the analysis of quantitative data to assess changes in mean confidence scores pre-and post-handbook. Qualitative analysis of participant feedback was also done, and key themes were analysed. Finally, in the ‘Act’ phase, the handbook was revised, and changes were made based on feedback to improve clarity and conciseness. Revised versions were implemented in subsequent cycles. The first PDSA cycle was conducted from December 2024 to March 2025, and the second PDSA cycle was conducted from April 2025 to July 2025.

Data collection

Pre- and post-intervention surveys incorporated both quantitative and qualitative components. Quantitative responses used a five-point Likert scale (1 = Not confident, 5 = Very confident) to assess self-reported confidence in performing key haematology-related tasks [9]. Qualitative feedback was analysed thematically to explore participants’ experiences and perceptions of the handbook’s usability and relevance.

The pre- and post-handbook surveys comprised eight structured questions, which formed the basis of the main quantitative analysis for this project. These assessed self-reported confidence in several domains, including preparedness for the rotation, confidence in documentation, ability to perform common ward tasks, confidence in requesting blood products and completing associated paperwork, interpretation of blood results and initiation of treatment, confidence in taking haematological histories, ability to complete discharge paperwork, and arranging follow-up after discharge. The full pre-handbook questionnaire is provided in Appendix A and the full post-handbook questionnaire is provided in Appendix B. 

## Results

PDSA cycle 1 (December 2024 - March 2025)

A total of six resident doctors were emailed the handbook. Three doctors responded to the pre-handbook survey (50% response rate), and two doctors responded to the post-handbook survey (33% response rate). Quantitative data were analysed using mean differences. Qualitative data underwent thematic analysis to identify the scale of the handbook’s impact and feasibility. The results of the first PDSA cycle are summarised in Table 1.

**Table 1:** Confidence Scores Before and After Guide Implementation in Cycle 1 TTO: To Take Out (discharge summary)

Domain	Pre-Guide Average Score	Post-Guide Average Score	Difference
How prepared did you feel for this rotation?	2.40	4.00	+1.60
Confidence in documentation during ward round	2.60	5.00	+2.40
Confidence with carrying out common ward tasks	2.80	5.00	+2.20
Confidence requesting blood products and completing paperwork	3.20	5.00	+1.80
Confidence interpreting blood results & initiating prophylactic treatment	2.20	4.30	+2.10
Confidence in taking a haematological history	2.80	5.00	+2.20
Confidence documenting discharge summaries and TTOs	2.60	4.70	+2.10
Confidence arranging follow-up post-discharge	2.80	5.00	+2.20

Across all assessed domains, there was a notable improvement in self-reported confidence scores following implementation of the handbook. The average increase in confidence ranged from +1.6 to +2.4 points on the five-point Likert scale. Overall, these results demonstrate a consistent positive impact of the handbook on resident doctor preparedness and confidence during the haematology rotation. 

PDSA cycle 2 (April 2025 - July 2025)

The handbook was sent over to three new resident doctors starting their haematology placement in April. All three resident doctors (100% response rate) responded to the pre-handbook survey, and two doctors filled out the post-handbook questionnaire (66% response rate). Following on from Cycle 1, quantitative data for Cycle 2 were analysed for mean differences, and a thematic analysis was done on participants' responses for qualitative analysis. The results of cycle 2 are summarised in Table 2.

**Table 2:** Confidence Scores Before and After Guide Implementation in Cycle 2 TTO: To Take Out (discharge summary)

Domain	Pre-guide Average Score	Post-guide Average Score	Difference
How prepared did you feel for this rotation?	2.17	4.00	+1.83
Confidence in documentation during ward round	2.67	4.75	+2.08
Confidence with carrying out common ward tasks	2.83	4.75	+1.92
Confidence requesting blood products and completing paperwork	2.83	4.75	+1.92
Confidence interpreting blood results & initiating prophylactic treatment	2.00	4.25	+2.25
Confidence in taking a haematological history	2.67	4.75	+2.08
Confidence documenting discharge summaries and TTOs	2.33	4.50	+2.17
Confidence arranging follow-up post-discharge	2.50	4.75	+2.25

The results from PDSA Cycle 2 demonstrated continued improvement in resident doctor confidence across all assessed domains following implementation of the updated handbook. The mean increase in confidence scores ranged from +1.83 to +2.25 points on the five-point Likert scale, indicating a sustained positive impact of the intervention. Overall, these findings confirm the handbook’s ongoing positive impact in enhancing junior doctor preparedness and confidence during the haematology rotation.

Qualitative analysis

Analysis of qualitative feedback from both cycles showed that resident doctors frequently struggled with discharge documentation and follow-up for patients. Additionally, most expressed apprehension in the management of haematological emergencies, such as neutropenic sepsis and tumour lysis syndrome. Following Cycle 1 completion, it was found that resident doctors relied mostly on help from registrars and consultants to manage ward tasks; however, with varying luck due to ward pressures and staff availability. Following cycle 2 completion, it was expressed that resident doctors routinely used departmental induction and ward posters to seek help.

Following the implementation of the handbook, respondents highlighted its clarity and ease of use as key strengths. Feedback from both PDSA cycles indicated that the handbook was particularly helpful in providing guidance on follow-up pathways and departmental processes. After the first cycle, one participant recommended maintaining regular updates to ensure accuracy of contact information beyond the QI project. Post the second cycle, residents reported notable improvements in confidence regarding ward tasks and interpretation of abnormal laboratory results. All participants indicated that they would recommend the handbook to subsequent cohorts. After each cycle, the handbook was revised to remove redundant content and enhance overall readability.

## Discussion

Strengths of this QI project were that it utilised a simple, easily producible, and accessible intervention, making it sustainable and easily adaptable for further development. Additionally, the PDSA methodology enabled an iterative approach, allowing for continuous refinement based on user feedback. A mixed methods approach was also employed with participant feedback, allowing for both quantitative analysis and thematic analysis from user feedback. This comprehensive evaluation provided a well-rounded understanding of the intervention’s effectiveness in improving induction quality and junior doctor preparedness.

This project, however, also had several limitations. The sample size was small, as data were collected from a single district general hospital and limited to the number of resident doctors rotating through the haematology department during the study period. The variable survey response rate between pre- and post-intervention cycles may have introduced response bias and limited the representativeness of findings. Furthermore, the short evaluation period, spanning only two PDSA cycles, restricted the ability to assess the long-term sustainability of the intervention or its potential impact on clinical outcomes. Future iterations of this project could benefit from multi-centre collaboration, larger sample sizes, and longitudinal evaluation to better assess the durability and generalisability of the intervention’s benefits.

The QI project was presented at a local departmental audit meeting following completion of the two PDSA cycles. Attendees, including consultants, registrars, and resident doctors, agreed that the project effectively identified a previously unaddressed gap in departmental induction. Haematology consultants provided suggestions for further improvements to the handbook, informed by observed ward practices, to enhance resident doctor effectiveness. It was unanimously recommended that the handbook be incorporated into future departmental inductions, with the specialty induction lead informed of its inclusion. Plans were conceptualised to integrate the handbook into the Trust intranet, facilitating access for resident doctors undertaking weekend or locum shifts without prior departmental experience. Regular updates are planned to maintain the handbook’s relevance and accuracy in accordance with departmental and policy changes. These measures aim to embed the project within routine departmental practice, thereby sustaining its impact on junior doctor preparedness and patient safety.

## Conclusions

This QI project addressed a gap in the haematology departmental induction process through the introduction of a structured digital handbook for incoming resident doctors. The intervention was associated with improved confidence in key clinical and administrative tasks and was positively received by both trainees and senior staff. Its simplicity, low cost, and adaptability support its continued use as a sustainable departmental resource. Integration of the handbook into the Trust intranet and formal induction programme is expected to maintain its impact and ensure accessibility for future cohorts.

## Appendices

Appendix A

**Table 3:** Haematology Induction Pre-Handbook Questionnaire

Question Number	Question	Response Options
1	Job Title	Free text response
2	Time into Current Haematology Rotation	Less than 1 week; 1–2 weeks; 3–4 weeks; 4+ weeks
3	How prepared did you feel for this rotation initially?	1 = Not confident, 5 = Very confident
4	How confident are you in documentation during a haematology ward round currently?	1 = Not confident, 5 = Very confident
5	How confident are you with carrying out common ward tasks?	1 = Not confident, 5 = Very confident
6	How confident are you with requesting blood products and required paperwork?	1 = Not confident, 5 = Very confident
7	How confident are you with interpreting blood results and initiating prophylactic treatment if needed?	1 = Not confident, 5 = Very confident
8	How confident are you with taking a haematological history currently?	1 = Not confident, 5 = Very confident
9	How confident are you with documenting discharge paperwork and completing TTOs?	1 = Not confident, 5 = Very confident
10	How confident are you with arranging follow-up for patients post-discharge?	1 = Not confident, 5 = Very confident
11	How many times since your rotation started did you require help with discharge paperwork and/or make mistakes during the process?	Never; 1–5 times; 5–10 times; 10+ times
12	What part of the discharge pathway and/or documentation do you struggle with the most?	Free text response
13	Where would you access important contacts for patient follow-ups?	Board by doctor’s desk; Senior staff members; Other FY1s/SHOs
14	How would you seek help for unfamiliar tasks during the ward round?	Senior help; Other junior doctors; Ward nursing team; Hospital hub
15	How were you made aware of departmental activities throughout the week?	Departmental induction; Email reminders; Other colleagues
16	Are there any areas you feel unprepared for in this rotation?	Free text response
17	Any tasks you encountered so far on this rotation you would like some more help with?	Free text response

Appendix B

**Table 4:** Haematology Induction Post-Handbook Questionnaire

Question Number	Question	Response Type / Options
1	Job Title	Free text response
2	Time into Current Haematology Rotation	Less than 1 week, 1–2 weeks, 3–4 weeks, 4+ weeks
3	How prepared do you feel for this rotation after accessing the guide?	1 = Not confident, 5 = Very confident
4	How confident are you in documentation during a haematology ward round post-guide?	1 = Not confident, 5 = Very confident
5	How confident are you with carrying out common ward tasks?	1 = Not confident, 5 = Very confident
6	How confident are you with requesting blood products and required paperwork?	1 = Not confident, 5 = Very confident
7	How confident are you with interpreting blood results and initiating prophylactic treatment if needed?	1 = Not confident, 5 = Very confident
8	How confident are you with taking a haematological history post-handbook?	1 = Not confident, 5 = Very confident
9	How confident are you with documenting discharge paperwork and completing TTOs?	1 = Not confident, 5 = Very confident
10	How confident are you with arranging follow-up for patient’s post-discharge?	1 = Not confident, 5 = Very confident
11	Do you think the guide covers the discharge pathway in sufficient detail?	Not too much information given, some detail was covered, good level of detail, too much information
12	What part of the discharge pathway and/or documentation did the guide help with most?	Free text
13	How useful was the guide in accessing information about departmental activities throughout the week?	1 = Not useful at all, 5 = Very useful
14	How useful was the guide in accessing additional resources for junior doctors?	1 = Not useful at all, 5 = Very useful
15	How likely are you to recommend this guide to other junior doctors rotating into the specialty?	1 = Very unlikely, 5 = Very likely
16	Any improvements to the guide?	Free text
17	Any tasks you encountered so far on this rotation you’d like included in the guide?	Free text
